# Enabling effective differentiated service delivery transitions for people on antiretroviral treatment

**DOI:** 10.1097/QAD.0000000000003826

**Published:** 2024-01-15

**Authors:** Lynne Wilkinson, Anna Grimsrud

**Affiliations:** a IAS –International AIDS Society; b School of Public Health and Family Medicine, University of Cape Town, Cape Town, South Africa.

**Keywords:** antiretroviral treatment, differentiated service delivery, disengagement, established on antiretroviral treatment, HIV, transitions, treatment literacy

## Introduction

Within the dynamic landscape of HIV treatment and care, differentiated service delivery (DSD) has gained significant traction with prominence within global guidance [1–3]. DSD places the individual at the centre, adapting services to meet their needs and expectations at a particular time while reducing unnecessary burdens for people with HIV and the healthcare system. It adjusts the service delivery building blocks – ‘when’ (frequency of treatment refill-only collection compared with clinical consultations), ‘where’ (locations of these services), ‘who’ (cadre providing the service) and ‘what’ (service package) – to accommodate population, clinical characteristics and context [4–7].

DSD models for HIV treatment fall into more and less-intensive DSD categories. Less-intensive models commonly increase the length of treatment refills provided and the spacing of required consultations with a clinician at the health facility. For adults, DSD for HIV treatment for adults is supported within global guidance [1,8–10], widely incorporated in national policy [11–17] and was accelerated in response to COVID-19, especially in east and Southern Africa [18].

Despite the scaling of DSD implementation, there is a disparity in coverage across different populations, highlighting the need for inclusive policies. To date, eligibility for less-intensive DSD models has been dependent on clinical stability, generally including a minimum time on treatment and evidence of treatment success [1,8]. Increasingly, national policies are broadening eligibility criteria for less-intensive DSD models to include children, adolescents, pregnant and breastfeeding women [12,19]. Implementation coverage for specific populations remains behind the scale for the general population. Concurrently, there is increasing recognition that clients starting treatment or struggling with sustained engagement, and those not yet established on ART, may also benefit from less-intensive DSD models [20].

With the scale of DSD for HIV treatment, there are an increasing number of movements into, out of and between less and more intensive DSD models. These movements between models have become known as ‘DSD transitions’. There is now a need to understand how to support successful transitions between service delivery models.

This commentary aims to address the necessary for DSD transitions, defining three categories of transition and emphasizing crucial enablers that require attention when revising DSD guidance and implementation.

## Discussion

**Fig. 1:**
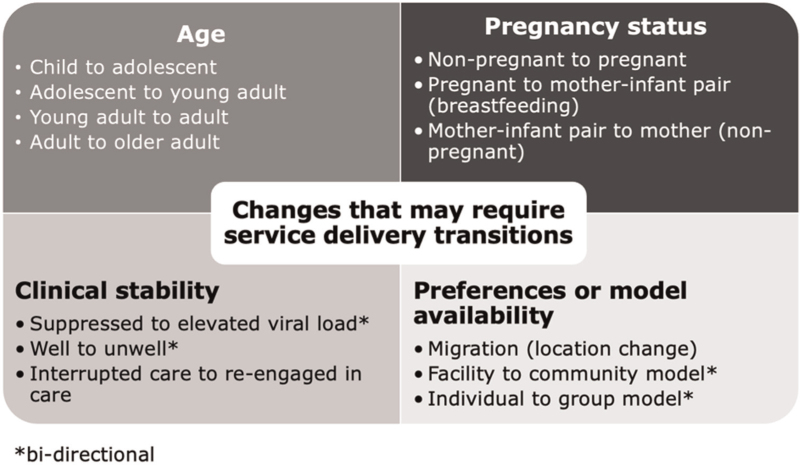
Changes that may require service delivery transitions.

HIV treatment demands a ‘life course’ approach, adapting services to changing needs and preferences during their ART journey because of factors like aging, pregnancy, clinical stability and personal circumstances (Fig. 1). These changes may necessitate a shift in the appropriate service delivery model.

Changes to age, pregnancy status, clinical stability and preferences or model availability are changes that may require service delivery transitions for someone who is accessing HIV treatment and care.

Transitioning between DSD models poses a heightened risk of treatment interruption. Individuals may not be prepared for the transition [21,22], face challenges in understanding or accepting it [23–26], or lose, or perceive to lose, a valued component of their care [24,25,27]. The health service may struggle to support an effective transition, leading to poor planning and inadequate management in the new model [23]. For example, a new health provider may not be expecting or ready for the referred client. Treatment refills may not have redirected to the new service location, or where DSD models provide longer drug refills, there may not be adequate drug supply.

Sub-optimal transitions elevate client dissatisfaction, increasing the risk of treatment interruption and long-term disengagement. There are strong associations between client dissatisfaction, disengagement and the reduced likelihood of re-engagement [28].

DSD transitions should be kept to a minimum to limit disruption and complexity both for the person with HIV and the health system. When transitions are necessary, clients should be supported by a health service enabled to facilitate a smooth transition.

DSD transitions fall into three main categories. First, ‘down-referrals’ from more intensive to less-intensive DSD models, second are transitions between less-intensive DSD models, and third, ‘up-referrals’ from less-intensive to more intensive models. Each category, along with essential client and health facility enablers, is elaborated upon below to facilitate successful transitions.

### Transitions from intensive to less-intensive differentiated service delivery models (‘down-referrals’)

Transitions from intensive to less-intensive DSD models, known as ‘down-referrals’, are critical. Eligibility for less-intensive DSD is often dependent on meeting established on ART eligibility criteria or re-establishing clinical stability after a period of illness, viral rebound, completing antenatal and/or postnatal care or a period of re-engagement (Table 1).

**Table 1:** Interventions to facilitate transitions from intensive to less-intensive differentiated service delivery models (‘down-referral’).

	Across population groups	Children	Adolescents	Re-engaging	Across populations groups
Reasons for transition	Assessed and qualify as established on ART for the first time				After clinical improvement or re-established on ART
Transition from (model)	Facility-based clinician-managed individual care				
Transition to (model)	Any less-intensive DSD model				
Enablers at the client level	Explain DSD model choices, detailing service frequency, location, provider) and package Offer enrolment (not automatic) Complete timely enrolment processes, including notifying new service providers and script submission (if external to health facility)	Enable enrolment into “family-centred” models for children, caregivers and family members (same building blocks). Transition may involve all willing family members or the child transitioning to the caregiver's less-intensive DSD model	+ Complete full HIV disclosure + Implement adolescent group DSD model/s and prioritize for offer + Assess individual readiness for transition + Develop a timeous transition plan with the adolescent + Assign a adolescent peer to support transition + Orientate the adolescent to any new location/peer group before the transition	+ At the re-engagement visit, communicate the follow-up visit schedule, including when eligible for multimonth dispensing (MMD) and assessment for less-intensive DSD. + Adhere to timeframes communicated	+ Assess for timely re-enrolment in less-intensive DSD (adhere to timeframes communicated) + Prioritize re-enrolment in previous model or same group/service provider, if preferred
Enablers at the health system level	Provide job aides detailing the mechanics of each less-intensive DSD model to support healthcare workers’ explanation to clients Include ‘down-referral’ service delivery transition enablers in national DSD operational guidance Establish clear DSD model enrolment and up-referral processes at facility level			+ Develop a national algorithm differentiating service delivery on re-engagement including eligibility for MMD and accelerated access into less-intensive DSD models + Provide facility-level training and job aides to support implementation	+ Adjust clinical ART stationary to identify current DSD model at every clinical visit (to support re-enrolment offer)

+ denotes additional enablers specific to certain populations groups. ART, antiretroviral therapy; DSD, differentiated service delivery.

#### Enabling down-referral after meeting ‘established on ART’ criteria

##### Across population groups

Ensuring smooth down-referrals involves effective communication of eligibility criteria and available models across diverse populations. Current gaps in communication often lead to transitions lacking explanation or choices for individuals, negatively impacting outcomes [24,25]. Enhancing DSD treatment literacy including eligibility details and model mechanics, can improve outcomes and foster increased demand for DSD, affording clients more options [20,23,24].

##### Among children and adolescents

Enrolling children in separate paediatric-only DSD models may disrupt family health visit schedules and negatively impact outcomes. Providing ‘family-cantered’ care, in the same less-intensive DSD model (i.e. ‘when’ – same appointment time, ‘where’ – location, ‘who’ – provider, and ‘what’ – receiving the same length of treatment refill), improves outcomes [29–33]. Where country guidance requires more frequent clinical reviews for a child ‘established on ART’, commonly in those less than 2 years old, every effort should be made to review the caregiver's preferred model to enable alignment of visit schedules and location.

For adolescents, supporting gradual independence in health management, introducing peer interaction and reducing visit burden, and offering ‘teen clubs’ has been shown to improve outcomes [34–39]. A timely transition into adolescent specific less-intensive models promotes retention, supported by readiness assessments, planning and orientation.

#### Enabling down-referral after re-engaging in care

Qualitative data of service delivery needs and preferences after disengagement and re-engagement highlights the importance of flexibility for clinical consultations and drug refills [40,41]. Accelerated access to a less-intensive model can aid sustained retention, eliminating health systems barriers associated with disengagement [20,42]. Clear explanations of available less-intensive DSD models and visit schedules are essential. Re-enrolment in a previously satisfactory model and leveraging established peer support networks are recommended strategies. Recent DSD guidance in Mozambique, South Africa, and Zimbabwe all facilitate accelerated access to less-intensive models for clients re-engaging in care [13–15].

### Transitions between less-intensive differentiated service delivery models (‘lateral moves’)

Transitions between less-intensive DSD models occur when individuals, while maintaining clinical stability, switch preferences from facility-based to out-of-facility or from individual to group models often due to age, completed postnatal care, a change in preference or circumstances like relocation. These transitions can be considered as ‘lateral moves’.

#### Facilitating age and pregnancy-related moves

Adolescents and postpartum individuals shifting from specific population adapted DSD models to general ART services, face challenges in care continuity [43–46]. To ease these transitions, transition preparation; including transition orientation, plans and readiness assessments, have been shown to improve outcomes after transition [21,47,48]. Gradual transition and transitioning groups or cohorts should be considered to retain the established, supportive peer network (Table 2). Gradual transition allows for elements of the new DSD model to be introduced within the previous model prior to the move such as longer drug refills, orientation to a new location or service provision and introductions to new service providers. For example, in South Africa, older members of youth clubs (located in youth specific clinics) were offered and supported to form their own adult adherence club at the adult ART clinic and transition together from adolescent to adult care [49,50]. Eswatini's 2022 national DSD guidance introduces ‘transitioning clubs’ specifically aimed at supporting transition from adolescence to young adulthood [51].

**Table 2:** Interventions to facilitate transitions between less-intensive differentiated service delivery models (‘lateral moves’).

	Across population groups		Children	Adolescents	Postnatal
Reason for transition	Change of DSD model preference	Transfer to new health facility	Aging into adolescence	Aging into adulthood	Infant >18 months old
Transition from (model)	Any less-intensive DSD model	Any less-intensive DSD model at previous health facility	Family-orientated DSD model	Adolescent specific DSD model	Postnatal DSD model
Transition into (model)	Any other less-intensive DSD model	Any less-intensive DSD model available at new health facility	Unaccompanied adolescent less-intensive DSD model	General adult less-intensive DSD model	
Enablers at the client level	Check on DSD model appropriateness and satisfaction at every annual clinical review Where there is a change in service delivery need or preference, explain the appropriate DSD model choices available Same as Table 1 – offer preferred model and complete timely enrolment processes	Where system is in place, explain how to change ART refill collection geographical location Include in transfer documentation the DSD model utilized prior to transfer Prioritize assessment for less-intensive DSD at transfer-in visit and offer immediately if eligible.	Same as Table 1 for adolescents – full disclosure, readiness assessment, transition plan, adolescent DSD models, peer-led case management for transition period and orientation visit Gradually transition by introducing new model building blocks into current model (for example, increasing refills or contraceptive care in the service package) Where possible, transition a group of children/adolescents together (i.e. cohort transitioning) For adolescents transitioning to adult care, prioritize offer of any available adult group model that could allow a group of new young adults to join or start a new group together.		Develop a timely transition plan with postnatal woman confirming their preferred less-intensive DSD within the general ART service and the timing of the transition. Provide mentor mother navigation/accompaniment to new service location, including an orientation visit Where possible, transition a group of postnatal women together (cohort transitioning) If direct transition to less intensive DSD is not possible, assess and offer DSD options at the first clinical consultation in the general ART service
Enablers at the health system level	Same as Table 1 with national DSD operational guidance to consider ‘lateral move’ service delivery transitions enablers	Set up simplified processes for geographical change to ART refill collection elsewhere in country	Facilitate facility level planning for transitions including providing readiness assessments, ensuring suitable DSD model availability, enabling gradual transitioning mechanisms, implementing cohort transitioning and new model orientation approaches		

ART, antiretroviral therapy; DSD, differentiated service delivery.

#### Enabling service preference changes between models within a facility

Changes in personal circumstances or satisfaction level might prompt a shift in HIV service delivery preferences. A change in home or work could result in a new location being more convenient. A new health condition need may require more frequent visits to the health facility making fast-track facility refill collection more practical than community collection. A person may experience inter-personal conflict in a group DSD model or have a negative experience with a service provider prompting a change in preference.

The offer of a less-intensive DSD model should not be a once off event. Health systems should support routine DSD model preference assessment, ideally at the annual clinical visit, and enable a person to request a model change. Uganda's clinical stationary requires, at each clinical consultation, the assessment of DSD-related categorization. Training for healthcare workers supporting DSD in Uganda emphasizes a ‘5As approach’ – **A**ssess the person's knowledge of DSD; **A**ssist the person to identify barriers to continued care; **A**dvise the person on appropriate DSD models; **A**gree with person on their DSD model of choice; **A**rrange for the person to receive ART drug refills and clinical management under their preferred DSD model [52].

#### Enabling lateral moves related to transfer to a new facility

Efficient systems supporting transfers between facilities are vital. Any transfer documentation should specify the model at transfer to facilitate immediate enrolment at the receiving health facility. Immediate enrolment, or at a minimum immediate assessment, for less-intensive DSD upon transfer supports uninterrupted care.

Broader systems that enable changes in ART refill collection points are also becoming increasingly critical. An example is South Africa's central chronic medicines dispensing and distribution (CCMDD) programme that enables the client, rather than the healthcare provider, to change their treatment collection point throughout a toll free number [53]. This mechanism remains largely unutilized and untested due to limited awareness.

### Transitions from less-intensive differentiated service delivery model to intensive differentiated service delivery models (‘up-referrals’)

Moving from less-intensive to more intensive DSD models becomes necessary when clinical circumstances change, requiring increased clinical management or if specific populations become ineligible for less-intensive DSD models (Table 3). For instance, pregnancy often necessitates transitioning to integrated antenatal and HIV care. These transitions are termed ‘up-referral’.

**Table 3:** Interventions to facilitate effective transitions from less-intensive to intensive differentiated service delivery models (‘up-referral’).

	Across population groups
Reason for transition	Clinical reasons or preference to integrate care or in terms of DSD policy ineligible for less-intensive DSD
Transition from (model)	Any ‘established on ART’ DSD model
Transition to (model)	Facility-based clinician-managed care (possibly integrated care)
Enablers at the client level	Clearly explain the purpose of increased clinical management and the timeline for re-enrolment assessment in less intensive DSD For those in group or individual community models with an established peer support network, offer choice to stay in DSD model alongside additional clinical care For clinically well individuals, continue to provide longer treatment refills, limiting clinical consultations and/or counselling sessions to those absolutely necessary Provide quality comprehensive clinical management to ensure service delivery change has added value (not only rescript and drug refill) Track and trace clients who miss follow-up clinical appointments
Enablers at the health system level	Implement quality assurance processes to support the provision of comprehensive care to people requiring more intensive clinical management Implement audit processes to review clinical management quality and assess the appropriate timing of less-intensive DSD re-enrolment

ART, antiretroviral therapy; DSD, differentiated service delivery.

#### Enabling up-referrals for increased clinical management

As highlighted, access to less-intensive DSD models is generally dependent on eligibility criteria such as no current illness, good understanding of lifelong adherence and evidence of treatment success in addition to a minimum of time on ART [1]. When individuals no longer meets these criteria, national DSD guidance often mandates up-referral for increased clinical management reverting to monthly clinical consultations at the health facility. This is often done without clear explanation, causing clients to perceive it as punitive, rather than as supportive [24].

Improving the explanation of the eligibility criteria upon model entry can facilitate smoother transitions later on. Healthcare providers investing time in clarifying the need for increased clinical management, along with a clear timebound pathway back to a preferred less-intensive model, could enhance transition experiences. There are also examples where a person experiencing an elevated viral load is supported, if preferred, to stay in their less-intensive DSD model (commonly a group model) with additional clinical consultations alongside [15]. National DSD guidance updates increasingly attempt to cushion up-referral transitions for clients clinically well with elevated viral loads by continuing to provide longer ART refills, especially for clients with difficulties getting to the facility [13–15,54].

## Conclusion

Service delivery transitions are an integral, but often neglected, component of DSD. They require increased attention as more people are accessing DSD models and may require supportive transitions as their needs change and evolve. DSD's central premise is one where the person is at the centre – and therefore transitions should be enabled when required, and minimized when unnecessary as they can be disruptive and complex both for the individual and the health system. Increasing DSD literacy and service delivery-related communication among and between healthcare workers and clients is key. DSD processes and health systems can and should enable effective transitions. National DSD operational guidance should include guidance on how to successfully facilitate service delivery transitions. Where such guidance supports population-specific models, the approach to transitioning out of the specific DSD model should also be outlined. With many service delivery transition points to consider, the transitions making the largest contributions to treatment interruptions or disengagement should be identified and support strategies developed and implemented. Evaluation tools to assess the quality and effectiveness of implemented transition strategies should also be considered and could include patient satisfaction surveys, retention rates, viral suppression outcomes, and cost-effectiveness analyses.

## Acknowledgements

Authors’ contributions: conceptualization (L.W. and A.G.), project administration (L.W. and A.G.), writing – original draft (L.W.), writing – review and editing ((L.W. and A.G.).

Other acknowledgements: We would like to thank Helen Bygrave for her inputs and role in the DSD and transitions session at the 24th International AIDS Conference (AIDS 2022). We also thank the presenters during this AIDS 2022 session including Stanley Ngoma, Cordelia Katureebe, Helder Macul, Lillian Mworeko and Kombatende Sikombe. Finally, we thank Lina Golob for her project management and administrative support.

Funding: L.W. and A.G. are supported by the Bill & Melinda Gates Foundation through INV-047567.

### Conflicts of interest

There are no conflicts of interest.
